# Hypnotherapy for irritable bowel syndrome: patient expectations and perceptions

**DOI:** 10.1177/17562848221074208

**Published:** 2022-02-15

**Authors:** Anne-Sophie Donnet, Syed Shariq Hasan, Peter J. Whorwell

**Affiliations:** Neurogastroenterology Unit, Wythenshawe Hospital, Manchester, UK; Hypnotherapy Unit, Wythenshawe Hospital, Manchester, UK; Neurogastroenterology Unit, Wythenshawe Hospital, Manchester M23 9LT, UK

**Keywords:** expectations, hypnotherapy, irritable bowel syndrome, perceptions

## Abstract

**Introduction:**

Numerous studies have shown that hypnotherapy (HT) is effective in irritable bowel syndrome (IBS) using traditional symptom severity end points. However, there is now interest in capturing the patient’s perception of their illness and treatment because what patients expect from their treatment may differ from that of their healthcare provider.

**Objective:**

To record patient perceptions and expectations of hypnotherapy as well as their symptom response.

**Methods:**

150 consecutive IBS patients (116 females, 34 males, aged 16–81 years) receiving hypnotherapy completed questionnaires recording IBS symptom severity, quality of life, noncolonic symptoms, anxiety and depression levels before and after treatment. Their expectations and perceptions of HT were also recorded, including a free text reflection.

**Results:**

121 patients (81%) responded to treatment consistent with our previous experience. Symptom severity scores, noncolonic symptoms, quality of life, anxiety and depression significantly all improved after HT (*p* < 0.001). Expectancy of an improvement with hypnotherapy was greater in those who did not respond to treatment (63%) than those who did (57%, *p* < 0.001). Scepticism and apprehension were common before treatment and replaced with enthusiasm afterwards. Free text responses after treatment were overwhelmingly positive. Patients also reported a variety of other benefits and even 20 of 29 symptom nonresponders (70%) still considered treatment worthwhile.

**Conclusion:**

Although initially perceived negatively, hypnotherapy improved symptoms and resulted in a wide range of additional benefits. Expectation did not necessarily influence outcome. Recording IBS symptoms alone does not fully capture the patient’s experience of treatment and needs to be considered in future research.

## Introduction

Irritable bowel syndrome (IBS) affects approximately 10% of the population and remains a challenging condition to manage.^1–3^ This is partly because the pathophysiology is not fully understood, although there is evidence that gastrointestinal motility, visceral sensitivity, the central processing of noxious stimuli and the gut microbiome may be abnormal in some, if not all, IBS sufferers.^4^ Furthermore, diet can play an important role as well as a variety of psychological influences.^4–6^ With such a wide range of factors involved, it is not surprising that finding a medication that can target at least some of them is challenging. Furthermore, it would be a bonus if an agent aimed at modifying gastrointestinal physiology could have an additional effect on psychological factors.

As an alternative approach to pharmacological treatment, our unit has been undertaking research into the possibility that hypnosis might not only improve psychological symptoms, which is a well-known benefit, but also have the capacity to modulate gastrointestinal physiology. We have developed the technique of ‘gut focused hypnosis’ (GFH) and have shown that it not only improves the symptoms of IBS but can also influence gastrointestinal physiology.^7,8^

Subsequent clinical trials by both our team and others have shown that GFH leads to a significant improvement in the symptoms of IBS, in both adults and children.^8–10^ In addition, it has been shown to be effective in other functional gastrointestinal disorders, such as functional dyspepsia^11^ and noncardiac chest pain.^12^ While the treatment response rate does vary between different studies, we have reported a fairly consistent figure of approximately 70% and in a recent audit of 1000 consecutive patients attending our service the figure was 76%.^13^

Our first trial of hypnotherapy in IBS was published in 1984^14^ and it is disappointing that this form of treatment has still not been made widely available, despite confirmation of efficacy by others and its endorsement in the United Kingdom by the NICE guideline on the treatment of IBS. Unfortunately, much scepticism and prejudice still surrounds the use of hypnosis especially for medical rather than psychological conditions^15–17^ and this may account for some of the lack of interest from the medical profession. It also likely that this attitude is shared by the general public and some patients.

In the context of IBS, the notion of the ‘patient’s perspective on treatment’ is very topical,^18^ especially as the Food and Drug Administration (FDA) has advocated the use of patient-reported outcomes (PRO) in order to assess drug efficacy.^19^ In light of the increasing interest in capturing the patient’s perception of their illness and especially their treatment, we have been recording patient perceptions and expectations of hypnotherapy and now report the results for the first 150 patients assessed.

## Methods

In all, 150 consecutive IBS patients (116 females, 34 males), mean age 46 years (range: 16–81 years) attending the hypnotherapy unit at Wythenshawe Hospital, Manchester over a period of 9 months, were asked to participate in the study. All patients met the Rome IV criteria for IBS and were divided into diarrhoea and constipation subgroups.^20^ They were asked to complete the usual questionnaires that we routinely collected before and after treatment. In addition, they were asked to complete a questionnaire about their perceptions and expectations of hypnotherapy at the end of treatment. The questionnaires were as follows.

### The IBS Symptom Severity Score^21^

This score consists of five items (pain severity, pain frequency, abdominal bloating, bowel habit dissatisfaction, life interference), each scoring up to a maximum of 100, the sum of which allows patients to be classified as suffering from mild (<175), moderate (175–300), and severe (>300) IBS. A score of less than 75 includes 95% of a non-IBS population and would be regarded as indicating remission in a patient with IBS. In order to assess response to hypnotherapy in percentage terms, the proportion of patients achieving a 50-point or more reduction in symptom severity was calculated, as it has been shown that such a reduction is indicative of clinically significant improvement.^18^ These patients were defined as responders for the purpose of further analysis. Response rates for the more demanding endpoints of a reduction in score of 100 and 150 points were also calculated. The individual component scores of the IBS Symptom Severity Score (IBS SSS) were also documented.

### Noncolonic Symptom Score^22^

This consists of 10 items [nausea/vomiting, early satiety, headaches, backache, lethargy, excess wind, heartburn, urinary symptoms, thigh pain, and aches and pains in muscles and joints (bodily aches)], each scoring up to a maximum of 100, the sum of which is divided by two to give a maximum score of 500.

### Quality-of-Life Score^22^

This consists of 15 items, which are scored on a 0–100 scale with a higher score indicating a positive response to a particular question, which is the opposite to the other questionnaires. For instance, a positive response to *‘how are you coping with problems’* would be *‘very well’* whereas a positive response to *‘how often do you worry’* would be *‘never’.* The 15 components were as follows: coping with problems, confidence and security, quality of sleep, feelings of irritability, frequency of worrying, enjoyment of life, feelings of hopefulness, physical wellbeing, relationships with others, maintaining friendships, feelings of inferiority, feeling wanted, feelings of helplessness, difficulty making decisions and enjoyment of leisure time. The sum of these 15 components was divided by three to give a maximum Quality-of-Life Score of 500.

### Hospital Anxiety Depression Questionnaire^23^

This consists of seven anxiety and seven depression-related questions each of which can be responded to on a scale of 0–3, giving a maximum score for either domain of 21. There is no single generally accepted cut-off score for the Hospital Anxiety Depression (HAD), although Zigmond and Snaith suggested 7/8 for possible and 10/11 for probable anxiety or depression and we have always chosen to use a value of 10 or above in our previous studies. Some authors have suggested slightly lower cut-off values,^24^ but these have not been universally accepted. Consequently, to allow comparison with our previous work, we have continued to use a cut-off of 10 in this study. In addition to using a cut-off, we also quote the mean scores for anxiety and depression, as we have done in the past.

### FDA recommended definition of a responder^25^

The US Department of Health and Human Services Food and Drug Administration Centre for Drug Evaluation and Research (CDER) has recently suggested that a responder should be defined as an individual experiencing at least a 30% reduction in their pain score following treatment. Consequently, the percentage of patients experiencing a reduction of 30% or more in their pain score derived from the IBS SSS was calculated.

### Expectations and Perceptions Questionnaire

The Hypnotherapy for IBS Patient Perception Questionnaire was designed internally before the beginning of the study. It consists of two parts:

Part 1 includes questions relating to the patient’s mind-set and perception of hypnotherapy before the start of treatment. This section also incorporates questions concerning the patient’s personal views about the hypnotherapeutic process prior to treatment and what factors may have influenced them, such as the Internet or the views of others.

Part 2 is designed to explore the patient perceptions of hypnotherapy at the end of the treatment. It investigates aspects such as the patient’s understanding about the nature of treatment, how effective he or she thought it was, their thoughts about the future and any indirect benefits to their daily life.

The questionnaire contains 21 open-ended questions, giving patients the opportunity to express themselves fully, to share their stories about the reality of their illness and to comment on the effects of hypnotherapy. These open questions encourage the patient to give, in their own words, any information they might feel is relevant even if it is about the negative aspects of hypnotherapy. Their unedited narratives were divided into themes with notable examples quoted in the paper and the remainder listed thematically in the supplementary information.

In order to generate quantitative data for the purpose of statistical analysis, more specific questions were asked. These were questions requiring either yes/no, single-word answers or answers in the form of numerical scales. Consequently, both qualitative and quantitative data were generated in this study. It was felt to be important to include real-life narratives from patients as well as quantitative data for statistical analysis.

### Hypnotherapy

All patients were offered up to 12 sessions of GFH, although some patients finished early because they felt they had reached maximum benefit. In these individuals, all questionnaires were completed when they finished treatment.

Hypnotherapy was delivered on a one-to-one basis by the same therapist. This involves an initial consultation in which the patient meets their therapist, who takes a history and explains the concepts behind GFH. The technique has been described fully elsewhere^8^ but consists of a brief tutorial about the pathophysiology of IBS and how the various putative mechanisms that have been implicated can be influenced by either the use of imagery or tactile means. During the next two to three sessions, hypnotherapy is induced through the standard means of eye closure, progressive muscular relaxation and standard deepening techniques. As the course of treatment progresses, more emphasis is placed on controlling gut function with the ultimate aim of enabling the patient to be ‘in control of their gut’, rather than their gut controlling them. The therapists are allowed some flexibility to alter their approach according to the patient’s symptomatology, but only superficial psychological issues are addressed, such as stress, anxiety and coping, as well as abnormal cognitions. Some patients do not seem to require 12 sessions of treatment to achieve significant improvement and we have recently published a paper showing that six sessions may be just as effective as 12 although some of the more severe cases do need additional sessions.^26^

### Statistical analysis

Patient data and quantitative results from the different questionnaires were entered into the statistical package SPSS 22. All variables were assessed for normality. Paired *T*-tests were used to compare the means of all individual components of the SSS, Noncolonic Score, Quality of Life Score and HAD questionnaire before and after hypnotherapy. The Chi-square test was used to compare responders *versus* nonresponders in their expectation of good outcome. Finally, the McNemar–Bowker test^27^ was used to identify any significant changes in the proportion of patients suffering from a certain classification of IBS severity (mild, moderate or severe). Given the high number of statistical tests carried out on this study sample, it is acknowledged that there is an increased risk of statistical significance being observed by chance. Hence, only results with *p* < 0.01 were considered as providing strong evidence of a significant difference. For data relating to patient perception, there were a small number of missing responses (<5%) for some items.

### Ethical considerations

Aside from the expectation and perception questions, all questionnaires included in this study are used routinely in the department for audit purposes. Consequently, it was considered that this study was a combined audit and service evaluation and therefore ethical review was not necessary. The study was assessed via the Medical Research Council and NHS Health Research Authority decision tool,^28^ which confirmed that additional ethical approval was unnecessary. Despite this, signed consent was obtained on all patients participating in this study.

## Results

### IBS SSS

**Figure 1.**
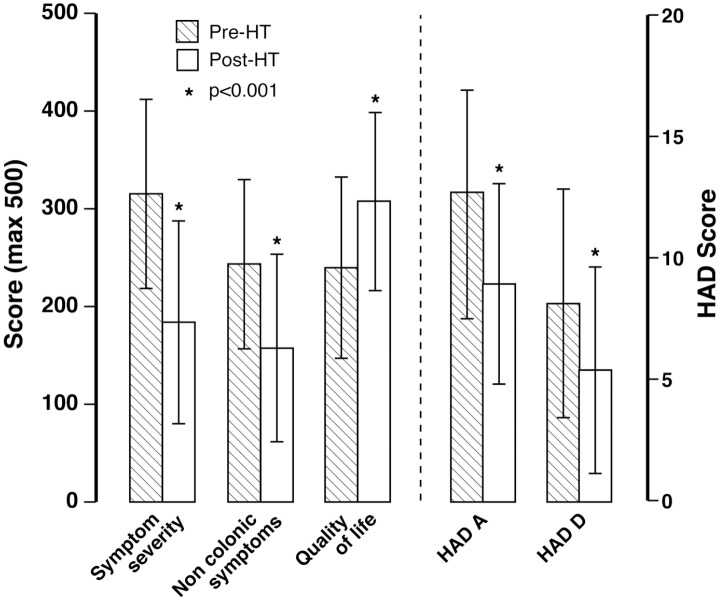
IBS symptom severity scores, noncolonic symptom scores, quality-of-life scores, anxiety (HAD A) scores and depression (HAD D) scores before and after hypnotherapy.

A total of 121 patients (81%) experienced a 50-point or more reduction in their total SSS, which is considered as being clinically significant (Figure 1). These patients were subsequently categorized as responders. Of these, 89 patients (60%) experienced a 100-point or more reduction in their total SSS score, and 59 patients (39%) experienced a 150-point or more reduction in their score. In addition, 63% of patients experienced at least a 30% reduction in their pain severity scores, which is the FDA’s latest definition of a responder in IBS.

Prior to treatment, the majority of patients (59.7%) were suffering from severe IBS as defined by their individual IBS SSSs; 33.7% had moderately severe IBS and only 9.7% were mild. Following hypnotherapy (HT), the majority of patients (51.3%) were mild, 32% moderate and only 16.7% were severe (*p* < 0.001).

Post-treatment, we found a significant reduction in the scores for: pain severity (pre-HT 52.7 *versus* post-HT 28.9, *p* < 0.001), pain frequency every 10 days (pre-HT 6 days *versus* post-HT 4 days, *p* < 0.001), abdominal bloating severity (pre-HT 55.0 *versus* post-HT 30.3, *p* < 0.001), bowel habit dissatisfaction (pre-HT 71.2 *versus* post-HT 41.8, *p* < 0.001), and interference with life (pre-HT 75.0 *versus* post-HT 46.8, *p* < 0.001), with the overall mean score falling from 315.2 to 183.9 (*p* ⩽ 0.001; mean (95% CI) change 131.3 (115.5, 147.1)).

### Noncolonic Symptom Scores

A significant reduction in the overall noncolonic scores was observed with the mean value falling from 243.5 pre-HT to 157.3 post-HT [*p* < 0.001; mean (95% CI) change 86.1 (73.0, 99.2); Figure 1]. This was also the case for the individual components.

### Quality-of-Life Scores

There was a significant increase in the total quality of life score from a mean of 238.4 pre-HT to 306.7 post-HT [*p* < 0.001; mean (95% CI) change 68.2 (55.3, 81.1); Figure 1]. All components of the Quality-of-Life score were significantly increased.

### HAD Scores

There was a significant reduction in mean anxiety scores (Pre-HT 12.1 *versus* post-HT 8.9, *p* < 0.001) and depression scores (Pre-HT 8.0 *versus* post-HT 5.3, *p* < 0.001; Figure 1). In addition, the proportion of patients classified as anxious according to our criteria (scoring ⩾ 10) decreased from 69% before treatment to 42% post-treatment. Furthermore, the number classified as depressed fell from 32% to 19%.

### Patient perceptions of hypnotherapy

Patients provided a large number of comments to the open-ended questions in the study. For the purposes of this article, a limited number of quotations about patient perceptions before and after hypnotherapy are included in Tables 1 and 2. However, for the interested reader, all quotations are displayed in the supplementary data.

**Table 1.** Selection of comments from patients about their feelings about hypnotherapy before treatment.

Attitudes of referring doctor	• ‘She (the referring doctor) was very sceptical about referring me to the clinic, I am unsure she even knew about hypnotherapy. I had to persuade her to refer me’. • ‘GP unaware of how hypnotherapy worked – referral made on the basis that previous tests eliminated other causes of my IBS’. • ‘The doctor was very unsympathetic about my symptoms. He reluctantly referred me, he didn’t understand how the months of being afraid of leaving the house due to near accidents were affecting me’.
Other people’s impressions	• ‘I’ve met/known about 5 people (that went through our hypnotherapy programme). Overwhelmingly positive – glowing results’. • ‘No one tried to put me off, didn’t tell many people. Know a lot of people that are sceptical, didn’t want negativity or ridicule’.
Patient’s expectations of the hypnotherapeutic process before treatment	• ‘I think that I felt so desperate for help that I was willing to give anything a go. I didn’t know what to expect but I was hopeful that it might help me’. • ‘Worried about long term effects. Very sceptical, tool for weak people. Concerned about losing control. Didn’t really connect to medicinal hypnotherapy, more saw it as gimmick’. • ‘Expecting everything from this treatment, possibly too much’. • ‘Any improvement would have been welcome. Almost a last-ditch attempt’. • ‘Didn’t know much but was open minded about it and willing to try anything that could help alleviate the horrendous symptoms I was experiencing’.

IBS, irritable bowel syndrome.

**Table 2.** Selection of comments from patients about their feelings about hypnotherapy after treatment.

Hypnotherapeutic Process	• ‘Relaxing, healing, beneficial, remarkable. You have to trust yourself, trust the process and trust the person doing it. It helps in other areas of life as well. (The hypnotherapist) presents as a friend and takes you on a journey. He is very skilful but in spirit of partnership i.e. not as an authority figure’. • ‘A treatment to be explored, an opportunity through relaxation techniques to take back some control, to be in control, to regain a sense of self-worth and positivity’. • ‘Many functions of our body are taken care of by our unconscious mind (i.e. breathing, pumping of blood, digestion etc) hypnotherapy is a means by which we can talk to and control our unconscious mind’. • ‘Well worth the effort. There’s much to gain with no risk. There’s nothing to lose but a little time’.
Effect of hypnotherapy on IBS symptoms	• ‘Overall improvement – the frequency of bowel movement, the structure of the bowel movement, more formed. Urgency decreased on the whole. More confident to go to places, still needs to know toilet facilities are available, less pain in stomach’. • ‘My symptoms definitely improved! My stomach pain was reduced immensely and I have finally slept through the night’. ‘Sickness/nausea virtually disappeared, pain much improved, bloating and distention lessened’. • ‘Without it (hypnotherapy) I would still be suffering from pain on daily basis’. • ‘Cramps reduced. Event based reactions reduced. Lower reliance on Imodium – near 100% reduction’. ‘I have been very dependent on a number of drugs especially pain killers. For the past weeks I have taken two Paracetamol twice a week, no codeine, two doses of Buscopan’.
Effects on quality of life	• ‘Life changing – I am able to plan nights out, eat different food and I generally feel so much better’. • ‘Depression – I am not on anti-depressant tablets anymore and this is because he (my hypnotherapist) helped me so much’. • ‘Yes. Less anxiety about looking for toilets when I am out of home. Diarrhoea improved still experiencing it but less frequently’.
Value of hypnotherapy	• ‘After being dismissed and ignored over 25 years, from many GPs and hospital consultants, not understanding how much it controlled and ruined my life, this worked so well it speaks for itself. This has changed my life’. • ‘Extremely valuable and life changing health treatment which I feel extremely lucky to have been able to access. Helped me in many ways, forms I did not expect, such as how I interpret and manage life events and how I can improve my IBS by understanding this’. • ‘A very positive, relaxing and uplifting experience, which has given me ‘my life back’ quite literally. A deeper understanding of my inner self and what I can achieve’. • ‘Very pleasant sessions more relaxed and always positive. Enjoyed attending, feel that the course of 12 sessions has given me back control of my IBS, life changing’. • ‘I’m always lost for words when trying to tell somebody how incredible hypnotherapy has been for me. Honestly, the best thing I could have done’. • ‘Positive, pleasant, extremely successful. I am now able to manage my condition without pointless drugs for IBS. In conjugation with the initial stripping back the diet and identifying key sources, food stuffs causing flare ups, my IBS is under control’.
Changes in perceptions	• ‘Sceptic – before Believer – now’. • ‘Gave me an even better understanding of what hypnotherapy is, and it’s not just about making people look silly. Also made me realise there is more than just medication that can treat things!’ • ‘Had low expectations that it could work. Now, not sure how it works but cannot hide the fact that it has’. • ‘Always believed it could be helpful, but now totally convinced of the important part it plays in my treatment regime’. • ‘You were kind enough to offer me therapy in order to help me. With this in mind and even though I was at first sceptical, I decided to embrace it and give it a chance. All I can say is that it was a good decision and the whole experience I have found to be most rewarding and helpful’.
Therapy downsides	• ‘I am not sure I understand how to apply this “few weeks” of positive improvements to a condition that I’ve suffered with for 6 + years. What if the worst symptoms recur?’ • ‘The daily practice, whilst this is important it’s very hard to dedicate 1 hour a day everyday’. • ‘I do not feel that some of the repetition of the hypnotherapy words were appropriate to me, I wished that it could have been more person centred and words specifically tailored to my symptoms’. • ‘I cannot yet bundle up my bad thoughts about pain and bowel movement. They are clear as ever. The symptoms are still with me. I am braver about things but not totally’.

IBS, irritable bowel syndrome.

### Patient perceptions of hypnotherapy before treatment

#### Attitudes at time of referral

In all, 62% of the cohort (93 patients) had not heard about the hypnotherapy programme before attending the IBS clinic at Wythenshawe Hospital. Of the 38% that had, more than half (39 patients) came specifically for hypnotherapy. Prior to visiting the IBS clinic, 28 patients (18.6%) had seen four or more different gastroenterologists and five patients (3.3%) had seen more than six specialists in relation to their illness.

Patients were asked about their impression of the explanation provided about hypnotherapy by the referring doctor at the time of referral. Several descriptions about IBS and the hypnotherapeutic process seemed to confirm many of the misconceptions about IBS, such as that the condition being ‘all in the patient’s head’. Some patients said that they simply did not receive enough information. However, few explanations given by the referring healthcare professional portrayed hypnotherapy as a worthwhile method to control their condition.

#### Factors influencing patient impressions

The majority of patients (91.3% representing 137 patients) did not know anyone going through our hypnotherapy programme for IBS. Eleven patients were acquainted with people that had gone through our service and their opinions were universally positive; 87.3% of patients (131 in total) did not experience any negative reactions from their relatives about hypnotherapy. However, 12% (18 patients) had been advised against hypnotherapy either by relatives, friends or, in one case, a gastroenterologist. This was usually due to the misconceptions surrounding the hypnotherapeutic process or IBS. Some patients did not even want to disclose any information about this treatment for fear of negative comments. Patients were asked in the questionnaire if they had ever received hypnotherapy prior to being referred to our service. 42 (28%) patients had received a previous course of hypnotherapy for issues such as anxiety or smoking cessation. Interestingly, 26 out of those 42 patients had found the course effective.

#### Patient perceptions

**Figure 2.**
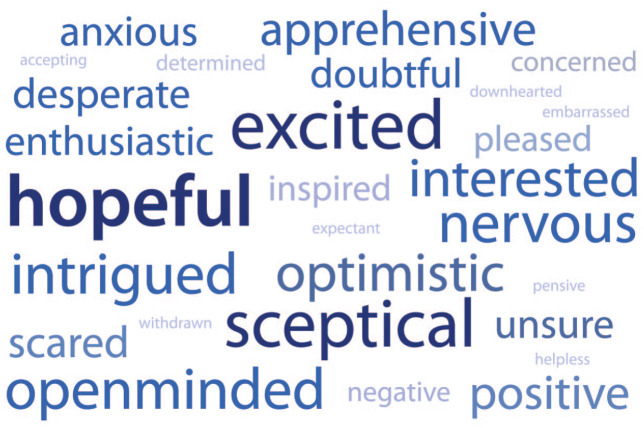
Word map of patient’s feelings before hypnotherapy. Greater prominence and size given to words that appeared more frequently.

To better understand the patient expectations before commencing hypnotherapy, they were asked to think of two words that best described how they felt prior to treatment. Figure 2 is a representation of their answers in the form of a word map, where greater prominence and size is given to words that appeared more frequently. A full list of responses can be found in the Supplementary data. Table 1 presents a selection of comments from patients about their feelings before hypnotherapy, with the remainder being available in the supplementary data Table S1.

Patients were asked to rate how effective they thought hypnotherapy was going to be on a scale of 1–10. For the purpose of assessment, a score of 1–5 indicated no efficacy and 6–10 indicated effectiveness of hypnotherapy.

In all, 60 patients (39.9%) thought that hypnotherapy was not going to be effective and 83 patients (55.3%) were anticipating that it would be helpful.

### Patient perceptions of hypnotherapy after treatment

**Figure 3.**
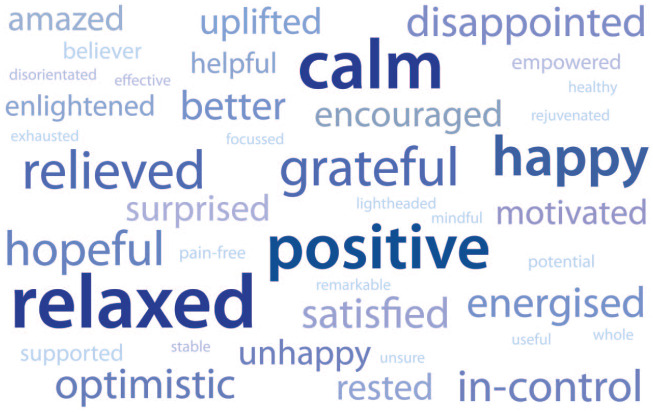
Word map of patient’s feelings after hypnotherapy. Greater prominence and size given to words that appeared more frequently.

Of the patients, 60.7% (91 in total) stated that their opinions concerning hypnotherapy had changed after the course of treatment. Many patients described how their initial sceptical views had been transformed into more positive and enthusiastic impressions about hypnotherapy. Patients were also asked to think of two words that best described how they felt after completing the treatment. The results are portrayed in a word map (Figure 3). Patients had more to say about the after-effects of hypnotherapy compared to their attitudes before treatment and Table 2 presents a selection of their comments with the remainder being available in the supplementary data Table S2. These are organized according to the question being answered.

### Alternative uses for hypnotherapy

Of the patients, 93.3% (140 patients) said they would use hypnotherapy for other reasons in their everyday life. These alternative effects, other than gastrointestinal symptom improvement, included being more relaxed, dealing with stress better, staying calm, sleeping better and controlling aches and pains. A full list is available in the Supplementary data Table S3.

In addition, patients were asked if they would recommend hypnotherapy to others based on their experience of treatment, with the strength of their recommendation rated on a scale of 1–10. All patients said they would recommend hypnotherapy to others, with 111 patients (74%) saying that they would score their recommendation as 10.

### Efficacy of hypnotherapy from the patient’s perspective

Patients were asked to rate how effective they thought their hypnotherapy had been on a scale of 1–10; 128 patients (85%) thought their hypnotherapy had been effective (scored greater than 6/10) and 21 patients (14%) felt it had not been helpful. However, when patients were classified as responders or nonresponders using the objective symptom measure of the IBS SSS, the number of responders was lower at 121 patients (81%). Consequently, it appears that more patients felt the treatment had been helpful using subjective measures compared to objective measures.

This is supported by the fact that in the nonresponder group, 20 patients (70%) thought hypnotherapy was still worthwhile despite the fact that they did not achieve a 50-point reduction in their SSS.

Interestingly, when patients were classified into responders and nonresponders using the IBS SSS, only 57% of responders had expected a good outcome compared to 62% of nonresponders (*p* ⩽ 0.001).

## Discussion

As previously stated, the adoption of hypnotherapy by mainstream medicine has been slow, despite strong evidence of its benefits and the sustainability of this effect. This is likely to be, at least in part, due to the scepticism that still surrounds the subject,^15–17^ with some detractors highlighting that clinical trials on this form of treatment cannot be truly double blind. However, the infallibility of the double-blind trial is now being questioned^29^ and may sway opinion.

In recent years, there has been a move to involve patients and the public in setting research priorities, following the recognition that there is not always agreement between researchers and research users on the agenda of future research.^30–33^ It is against this background that this study aimed to assess how patients with IBS perceive the subject of hypnotherapy before and after treatment.

Compared to baseline, the patients in this study experienced significant improvements in IBS symptom severity, noncolonic symptomatology, quality of life and psychological status. The magnitude of these changes was very similar to that observed in our larger previous publications,^13^ so it seems reasonable to conclude that the patient group included in this study is reflective of IBS patients in general, although they did all come from the same centre.

With regard to patient perception of hypnotherapy before treatment, it is clear that scepticism is not just confined to the medical profession. Consequently, if patients, even when they are suffering from severe symptoms, have this preconception, they are unlikely to consider this form of treatment unless they visit a unit which offers hypnotherapy as part of their therapeutic options. Another striking observation was the change in attitude to hypnotherapy following treatment, even when unsuccessful, with no negativity remaining.

Despite the fact we have published widely on the subject of hypnotherapy, it was disappointing to find that nearly two-thirds of patients had not heard of the unit. This might be, at least in part, because general practitioners and referring clinicians are not aware of this type of treatment or question its efficacy. Some patients had been actively discouraged from trying hypnotherapy by others and in one case by a gastroenterologist. This latter finding serves to further highlight the amount of prejudice that still surrounds the subject of hypnosis.

Expectation is often raised as a possible confounding factor in the interpretation of the results of clinical trials, particularly those concerning behavioural treatments. It is anticipated that patients who are more enthusiastic about a particular treatment would tend to do better than those who are less so.^34^ However, this is not consistent with our results, where we in fact found the reverse: 57% of responders expected treatment to be successful, compared to 62% of nonresponders.

One striking observation about the effects of hypnotherapy in IBS, in both this and previous studies, is that as well as relieving the abdominal symptoms of the condition, it also improves noncolonic symptoms, such as backache, lethargy, and bladder symptoms, as well as psychological symptoms such as anxiety and depression scores.^13^ This is in contrast to many pharmacological approaches, which tend to only improve symptoms to which they are targeted, such as abdominal pain in the case of antispasmodics.

Using the traditional outcome measure of a reduction in IBS symptoms, 19% of patients in this study would be classified as treatment failures. However, when asked directly, 70% of this group still said they felt that they benefitted from hypnotherapy. Furthermore, it is noteworthy that the majority of patients in this study also reported a variety of benefits in addition to relief of their IBS symptoms. These included the ability to cope with a range of other features of their illness such as the day-to-day challenges of everyday life, better sleep and the ability to relax. This capacity of hypnotherapy to improve so many other aspects of their lives presumably goes some way to explaining the observation that patients classed as treatment failures still reported that they benefitted from hypnotherapy. Furthermore, when asked if only one of their symptoms could be eliminated, patients not infrequently name a noncolonic symptom rather than a traditional IBS symptom. Consequently, the capacity of hypnotherapy to also relieve noncolonic symptoms may also help to explain their enthusiasm for the treatment when their IBS symptoms do not improve.

These results suggest that assessing the perception of a treatment such as hypnotherapy gives a more complete picture of a patient’s response rather than recording their gastrointestinal symptoms alone and better captures their desired outcomes. This needs to be accounted for when designing future clinical trials, especially of behavioural approaches, where a more global response to treatment might be expected. This reflects that what patients want from their treatment may not necessarily be what trial designers think they want.

In conclusion, this study confirms the considerable benefits of hypnotherapy in patients with IBS, both on symptom-based outcomes but also from a more holistic perspective, such as improvements in quality of life, depression and anxiety levels. It also highlights that scepticism is not confined to the medical profession and that there remain significant barriers to the widespread adoption of this highly effective treatment. However, there is some recent evidence from the United States that attitudes may be starting to change.^35^

## Supplemental Material

sj-docx-1-tag-10.1177_17562848221074208 – Supplemental material for Hypnotherapy for irritable bowel syndrome: patient expectations and perceptions Supplemental material, sj-docx-1-tag-10.1177_17562848221074208 for Hypnotherapy for irritable bowel syndrome: patient expectations and perceptions by Anne-Sophie Donnet, Syed Shariq Hasan and Peter J. Whorwell in Therapeutic Advances in Gastroenterology
