# Supplementary material for: Hypnotherapy for irritable bowel syndrome: patient expectations and perceptions
Source: Ther Adv Gastroenterol. 2022 Feb 15;15:17562848221074208. doi: 10.1177/17562848221074208 (PMC8859690; doi:10.1177/17562848221074208)
Supplement: sj-docx-1-tag-10.1177_17562848221074208 – Supplemental material for Hypnotherapy for irritable bowel syndrome: patient expectations and perceptions [file sj-docx-1-tag-10.1177_17562848221074208.docx]

# Supplementary Data

As explained in the paper, patients provided a large number of comments for the narrative part of the study. For the purposes of this study, a limited number of quotations about the patient perceptions before and after hypnotherapy are included in the paper. The remaining quotations are available in this supplementary section for the interested reader and are organised in the same way as the body of the paper itself.

# Patients’ Perceptions of Hypnotherapy before Treatment

## Attitudes at time of referral

“Because medically nothing is ‘wrong,’ they said it can help if it is in the mind and to think differently about IBS.”

“[After initial explanation] I now believe that it's in the mind and hypnotherapy helps you to control your illness.”

“She [the doctor] said she was happy to refer me as none of the tests she had done previously had shown any problems.”

“There seemed to be limited knowledge of the process technique.”

“Not sure she [the doctor] knew much about hypnotherapy.”

“My GP wasn’t informed until I outlined the treatment.”

“She [the doctor] didn’t. She was very sceptical about referring me to the clinic, I am unsure she even knew about hypnotherapy. I had to persuade her to refer me.”

“Consultant doesn’t believe in IBS.”

“GP unaware of how hypnotherapy worked – referral made on the basis that previous tests eliminated other causes of my IBS.”

“Very positive, encouraging. How patients had benefited. Explained how it helped with symptoms and explained about the links between gut & mind.”

“That the results have been good/positive.”

“Explained the research of Prof Whorwell and the benefits patients had after receiving hypnotherapy.”

## Factors influencing patient’s impressions

“I’ve met/known about 5 people [that went through the hypnotherapy programme]. Overwhelmingly positive - glowing results.”

“From an acquaintance who had previously been treated. They were very positive about it.”

“Word of mouth – a friend of a friend experienced reduction in IBS symptoms after a course of hypnotherapy.”

“My mother had a hypnotherapist-friend and suggested I’d try it as a form of therapy to cope with my dad passing away/puberty when I was a teen.”

“Some said i.e. boyfriend/male family I would end up suddenly making noises when I heard a certain word. Linked to a magician’s hypnotism.”

“My friends were very sceptical when they knew about it [the hypnotherapy treatment].”

“The consultant at the hospital refused three times to give me the referral requested to be seen by Prof Whorwell. I could not get a referral; my consultant dismissed my request so I wrote to Prof myself.”

“I read about it, the GP did not take my symptoms seriously. The usually dismissed as anxiety other times they gave me medication as though it is for stomach problems not bowel.”

“My husband – he said its mumbo jumbo but then he doesn’t understand alternative ideas and complementary medicine.”

“Neighbour and friend said: ‘That won’t do you any good, what do you want that for?’”

“My husband! Doesn’t understand how it can help!”

“Some of my friends only knew about people being hypnotised on shows and so they were making jokes about it.”

“Yes my mom. Religious reasons.”

“A psychiatrist - was not enthusiastic about hypnotherapy.”

“No one tried to put me off, didn’t tell many people. Know a lot of people that are sceptical didn’t want negativity or ridicule.”

“My GP – said he didn’t think it would make a difference.”

“No, though one of the consultants I initially saw was somewhat luke-warm about the idea…”

“Work colleagues – ‘bit ridiculous hypnotherapy for a dodgy stomach’. Demonstrates a lack of understanding of both IBS and hypnotherapy.”

“The doctor was very unsympathetic about my symptoms. He reluctantly referred me saying my symptoms were abhorrent, he didn’t understand how the months of being afraid of leaving the house due to near accidents were affecting me.”

“Other scientists at work – ‘I may be washing my money!’”

“It [reading about hypnotherapy] was positive and helped me alleviate some of my fears/concerns. I felt I would be able to undertake the hypnotherapy course. I had some doubts and it helped me resolve them.”

“I was sceptical about the actual process but very keen to try based on what I had read.”

“I thought that the idea and evidence that hypnotherapy could influence the gut directly was marvellous (I read this on your website).”

“As they say, never believe what you read in the press! Made me think it was all a bit of a showboat.”

“I’ve read that it works wonderfully for some people whereas some people are unable to grasp the technique.”

“I had it privately previously and it didn’t work [...] Didn’t do anything. Lots of interruptions.”

“Paid privately in Bristol, same reasons 8 sessions – no improvement.”

“Having tried hypnotherapy before I was optimistic that it would be effective.”

“I had previous successful treatment with hypnotherapy for smoking cessation. Extremely effective never had need or want to smoke since.”

“Hypnobirthing – powerful way to cope with labour + birth.”

“[Having hypnotherapy in the past] Allowed me to deal with long term self-esteem issues and uncover things gently.”

“I used it [hypnotherapy] before so I expected it will work.”

“Yes it [previous hypnotherapy] played a very calming influence on me when I had a fear of flying.”

“Hypnotherapy [previously] introduced me to my unconscious mind.”

“It [previous hypnotherapy] was effective because I feel that I may have formed habits (anxiety responses) that shaped actions and led to situations that were not constructive e.g. unnecessary comfort eating.”

“It [previous hypnotherapy] was privately, for grief counselling. It was effective because it gave me/my mind the tools to cope. I did not want to use antidepressants.”

“I saw a therapist for my back pain (CBT), He gave me a relaxation tape to listen to as part of the treatment. It wasn’t hypnotherapy but it did repeat – relax relax and did help me.”

“I had private hypnotherapy after I was getting flashbacks from a car accident on the motorway. It was very effective and I was pleased with results.”

“Just a few sessions [of non-GFH] for insomnia/waking up at night. Good at the time but not lasting. I feel like this current approach is more lasting/sustainable.”

“No, it [non-GFH] wasn’t as effective as those sessions. It was for other reasons that I was having previous hypnotherapy.”

“It [non-GFH] was effective but only short term. This course seems to have “stuck”.”

“It [non-GFH] wasn’t as strong as the hypnotherapy practiced here and the results didn’t last.”

“It [non-GFH] was less effective – it was another issue and I only had 2 sessions with no recording to listen to in between.”

## Patient’s perceptions of the hypnotherapeutic process before treatment

“I’ve tried ‘everything’ so was happy to try this regardless of GP/consultant view.”

“Because nothing has helped my condition so I had to explore other options.”

“Some research suggests it works, final thing to try […] last option.”

“A last attempt at sorting my gut out.”

“When first mentioned to me, I was willing to give it a go because I was desperate to get my life back on track, but I wasn’t hopeful, just wishing it worked, which it did.”

“It was clear from the book [by Prof Whorwell] that he knew precisely what problems are caused by IBS. First time in 19 years that I found someone who didn’t see IBS as a product of my imagination.”

“The NHS tests I had before seeing Prof Whorwell were: colposcopy, gastroscopy, stool tests, blood tests, ultrasound – abdomen and pelvis, MRI scan of the small bowel, bile acid test, SIBO hydrogen breath test. I was also initially sent down the gynae route for scans of the ovaries and blood tests due to the sudden change in bowel habit and my age. All tests came back as “normal”.”

“I was very open-minded. I thought it may work for some and prepared to have a go.”

“I was open to hypnotherapy having experienced other forms of non-invasive holistic therapy and found them beneficial.”

“I was very open minded. I think anything that will impact on my symptoms will be positive.”

“I think that I felt so desperate for help that I was willing to give anything a go […] I didn’t know what to expect but I was hopeful that it might help me.”

“Fairly open minded, keen to try this approach in conjunction with medication.”

“I hoped that the treatment would provide a regimen that would help me to deal with the restraints that IBS puts on a sufferer’s life.”

“Some reservations but welcomed any intervention that would help.”

“I felt fairly positive about it and was willing to try anything to help alleviate my symptoms.”

“Was unsure what the session would be like and thought it wouldn’t have massive effects […] It was a case of trying and see.”

“Unable to see how it would help my IBS.”

“I was frightened in case I didn’t know what I was doing.”

“Slightly uncertain but desperate to try.”

“Didn’t have much optimism on hypnotherapy thinking it was all in their minds. I couldn’t understand how relaxation would positively affect my pain.”

“Unsure as I never experienced it. Wasn’t confident it would work for me.”

“I was open minded. I knew there was proof that it worked but I assumed that it didn’t work on everybody. I really didn’t know what to expect as far as each session was concerned. So a little apprehensive.”

“Know very little about it. Fear my receptiveness for hypnotherapy.”

“Due to having tried years of different treatment for my IBS I was a little doubtful that hypnotherapy would work for me.”

“Worried about long term effects. Very sceptical, tool for weak people. Concerned about losing control. Didn’t really connect to medicinal hypnotherapy, more saw it as gimmick.”

“I am aware it’s about mindfulness, training to think about here and now rather than regretting about the past or worrying about future. I think this is going to be a challenge as I do have a very imaginative thought process.”

“I’ve never been before but my husband had used it previously, […] didn’t work for him so it was more like a waste of time as I thought it wouldn’t work for me either.”

“Expecting everything from this treatment (possibly too much).”

“To have a normal life.”

“An ability to take back control of my life in general.”

“My expectations were very high that this treatment would work and help improve my symptoms.”

“Hopeful. All else had failed. I wanted to believe in it.”

“I was sceptical but willing to try anything that would help with my IBS also it is important to keep an open mind about new things. I also had not understood how mind and stomach are linked.”

“Was hoping to feel a lot better than I have been feeling for years.”

“I don’t really know was hoping for a cure.”

“Relief from pain and bloating. To be able to sleep at night without being woken up constantly with pain. To get my life back and to look forward to things. Relief from worry over less important things.”

“I had tried lots of alternative medicines and nothing had ever helped my bowels only movicol.”

“To control the IBS symptoms of lower abdominal spasms and give me confidence in my ability to control my bowels.”

“Easing of symptoms. Not a cure.”

“More normal bowel habits/movement. A calmer abdomen. Less tightness and bloating.”

“No light at the end of the tunnel. Pain was there and controlling me.”

“Immediate stuff. I wished it would magically disappear.”

“At the best, an improvement on my condition. At the worst, no improvement.”

“Any improvement would have been welcome. Almost a last-ditch attempt.”

“Didn’t know much but was open minded about it and willing to try anything that could help alleviate the horrendous symptoms I was experiencing.”

“I will give anything a try to sort my IBS. Wasn’t sure what it would involve but happy to try.”

“I thought that I would be put to sleep by some magical watch before experiencing treatment.”

“Rating my organs in order of importance my brain is number 1. Do not like the idea of anyone messing with my brain.”

“I hadn’t realised hypnotherapy was a validated NHS treatment.”

“Only knew it as a regression therapy or as a tool for stopping smoking or assist with birth.”

“Thought it wasn’t really something offered by the NHS, like an alternative therapy or holistic treatment – not ground in science.”

“I didn’t think it would work as it has and I didn’t think I would have engaged with hypnotherapy as much as I have.”

“Thought it would be not much help, just mumbo jumbo. Found out it was all totally different, not like trash on telly.”

# Patients’ Perceptions of Hypnotherapy after Treatment

As explained in the study, after hypnotherapy, patients were found to be more talkative when answering questions set out for them in the questionnaire, and indeed, there was no shortage of words that patients expressed. These quotes are classified into sections depending on which question they were referring to in the questionnaire.

## Hypnotherapeutic process

“A very relaxing treatment that helps you to connect with yourself and focus on your condition and try to mend it as to make it better.”

“Relaxing, healing, beneficial, remarkable. You have to trust yourself, trust the process and trust the person doing it. It helps in other areas of life as well. [The hypnotherapist] presents as a friend and takes you on a journey. He is very skilful but in spirit of partnership i.e. not as an authority figure.”

“A non-invasive, painless technique which may help your condition, dramatically.”

“A very relaxed experience. It works even if you don’t believe in it before you start treatment.”

“Relaxing and a time to think about yourself/look after yourself. Worthwhile to help sooth and reset.”

“Meditation-max – targeted relaxation.”

“Deep relaxation that you in a trans state making you more susceptible to suggestions that can help change your life.”

“Relaxing, calming, kind, natural, non-invasive, respectful of mind and body.”

“A deep state of relaxation but it needs you to practice to improve.”

“Means of gaining control of your body and functions, a way to relax. Calming coping methods and means of reducing anxiety and stress. Helping me with pain relief and control.”

“A way to help you learn to cope and manage your illness and stress without more medication.”

“A treatment to be explored, an opportunity through relaxation techniques to take back some control, to be in control, to regain a sense of self-worth and positivity.”

“It’s the facility to unburden your horrible symptoms to a professional, who then talks to your brain about how best to resolve the imbalance.”

## Effect of hypnotherapy on IBS symptoms

“Pain - learned to manage it and control the feeling. Uncontrolled bowel movements - retrained my brain/gut/stress through CDs. Now able to leave home with ease.”

“All symptoms have improved, some more than others. Much greater control over emptying my bowels, less pain, much reduced fear of embarrassing situations and losing control of bowel movements.”

“Bloating, pain, nausea, discomfort, reactions after eating have improved particularly in weeks 8-12. My concern is this might be a temporary positive blip in the context of 6+ years of discomfort.”

“Diarrhoea has stopped and digestion slowed down.”

“I do not have as much bloating, spasm and open my bowels more frequently with more comfort also less movicol.”

“Without it [hypnotherapy] I would still be suffering from pain on daily basis.”

“Bowel movements have decreased from 7-8 times a day to 2 to 3. Greater control so not rushing to the toilet.”

“Symptoms have some improvement but better control and mind-set which I think helps symptoms too.”

“Sickness/nausea virtually disappeared, pain much improved, bloating and distention lessened.”

“No accidents since.”

“Yes still might get the odd pain but it is more manageable and less painful.”

“Stomach improved. Without a doubt it helps and still helping to the point I feel I could stop my pain altogether with practice.”

“Yes it helps me to relax and manage my stress levels which results in me being able to manage my lower abdominal pain.”

“Yes, pain discomfort, bowel movements, reduced stress.”

“My stomach pains and bloating have improved overall. They haven’t completely disappeared but I feel like I understand the symptoms more.”

“Yes. My diarrhoea has almost completely stopped and I rarely experience pain in upper left back(…) I still get bloated particularly in the mornings but I still feel even these things have improved.”

“Yes, very slow to see any improvement to start with but then good results.”

“Relieving acid reflux, I am now able to control it more through all the techniques that I have learned.”

“Yes, very few episodes of reflux, GORD now in control.”

“Yes, bloating, constipation, diarrhoea, pain, cramps, back pain, migraines all improved greatly.”

“Yes. I had bloating and abnormal bowel movements. I now only occasionally get bloating and have normal bowel movements. Yipee!”

“Yes – less bloating (virtually none, not at all), less upper and lower abdominal pain – only experienced when eating foods that trigger these symptoms.”

“Less pain, sleep improved, constipation much less.”

“Less wind, much less bloating, distention, no more tummy pains, more regular bowels.”

“Overall improvement – the frequency of bowel movement, the structure of the bowel movement, more formed. Urgency decreased on the whole. More confident to go to places, still needs to know toilet facilities are available, less pain in stomach).”

“All my symptoms have improved – bloating, constipation, bleeding, tiredness.”

“Yes, I was suffering with constipation and bloating and these have definitely improved. It has also helped me with stomach upsets where I have been able to calm my stomach down and put off going to the toilet.”

“Anxiety – decreased greatly, stomach pains less severe and less frequent, sickness almost non occurring, lethargy much less frequent.”

“My symptoms definitely improved! My stomach pain was reduced immensely and I have finally slept through the night. My treatment has helped my anxiety helping me to relax and release tension.”

“Symptoms have not improved however if I’m in a situation where I have time and a private space to relax e.g. at home in 15-30 minutes or so I can reduce the pain.”

“Cramps reduced. Event based reactions reduced. Lower reliance on Imodium – near 100% reduction.”

“On medication for the rest of my life but controlled dosages.”

“I have been very dependent on a number of drugs especially pain killers. For the past weeks I have taken two paracetamol twice a week, no codeine, two doses of buscopan.”

“Adrenaline decreased. Laxative use reduced – it helps removes working anxiety in which I hope to poo and therefore don’t, overall more confident.”

## Effects on quality of life

“Yes. Less anxiety about looking for toilets when I am out of home. Diarrhoea improved still experiencing it but less frequently.”

“They have, mostly my depression and anxiety – this link to my IBS as with me being more calm, I am not in as much pain and don’t go to the toilet as much.”

“Depression – I am not on anti-depressant tablets anymore and this is because [my hypnotherapist] helped me so much, bloating – although I need to improve my diet much more, pain – always helped me in ways to calm my bowels and therefore I wouldn’t be in so much pain, smoking – I’m smoking less.”

“Life changing – I am able to plan nights out, eat different food and I general feel so much better.”

“Frequency of bowel movement have reduced, better control of panic like thoughts while commuting.”

“IBS did not really improve as I have other illness – pemphigus. When I have a flare up I take more steroids and this does not make my stomach better. It did however help me to relax I am much more calm in stressful situations.”

“I had very loose stools with great urgency when I had to go to the toilet. Now I can calm myself down a lot of the time and change my diet.”

“Yes I can control my IBS better now. My panic feeling when I go out isn’t as much and I don’t take as much loperamide.”

“The way I cope when feeling stressed/overwhelmed has made my symptoms much more manageable if I put the work in. Over outlook on situations has me more confident, less stressed and give me tools and opportunities to manage and therefore my overall symptoms decrease.”

“Positive experience and I will take the relaxation techniques and use then forever.”

“My bowel habit hasn’t really improved but I have benefited significantly from the relaxation and the general advice provided by [the hypnotherapist].”

“My symptoms have not improved greatly, but I have learned to calm myself down and I can relax a bit more than before. I feel that stress contributes greatly to my condition, I am still learning to think positively.”

“As the treatment progress felt more relaxed and trusting and was impressed with effects of relaxation.”

“Very positive. I felt I can speak honestly to [the hypnotherapist]. He listens well, he is reassuring. I don’t feel judged (friend and family have often not understood the effects of IBS has had on me). This made me suppress it more and I am sure aggravated my self-confidence.”

“Not necessarily improved but now know how to manage them when bad.”

“I don’t think my IBS has improved – but my reaction to it has become more positive. I am less scared and more positive. I now have permission to go and lie down and relax.”

“I know for that hour every Tuesday its about me and my health and wellbeing. I enjoy the deep relaxation and the positive effects it has had on IBS. I have the tools necessary moving forward to practice and improve all the techniques (my hypnotherapist) has given me.”

## Value of hypnotherapy

“Like a form of relaxation to train the mind and body to think differently.”

“A life changing experience that can help positively alter mind-set to deal with struggles of life.”

“Increase understanding of how my body responds to my mind, valuable tool to learn deep relaxation which allows my mind and body to heal.”

“Amazing unbelievable. That to work on the mind helped control/stopping the pain.”

“Many functions of our body are taken care of by our unconscious mind (i.e. breathing, pumping of blood, digestion etc) hypnotherapy is a means by which we can talk to and control our unconscious mind.”

“I would reassure them that it’s not like stage hypnotism! I would tell them it’s a relaxing way of gently changing your mind-set.”

“Fantastic – a moment to focus on yourself and work on yourself. Hypnotherapy has made me realise how my mental/emotional state affects my physical state.”

“Like a mental massage.”

“Wonderful. Every session completely focussed on one aspect of my problem. Made me feel much more positive. Relaxed and less anxious. Would recommend to anyone if offered.”

“Well worth the effort. There’s much to gain with no risk. There’s nothing to lose but a little time.”

“My cure and control of IBS, an extremely positive and pleasant experience!”

“Unusual experience. Often uncomfortable due to me not opening up – interesting and effective.”

“Good – not always enjoyable – experienced some physical resistance to treatment to begin with, therapist easy to talk to especially when clarifying aspects of method.”

“I would say to give it a try, I found it useful and definitely beneficial.”

“Mind-blowing” How amazing the brain is, how much simple exercises can make a difference in your wellbeing – absolutely worth considering!”

“In terms of IBS specific hypnotherapy it was brilliant and very effective. It was worth waiting a year for it.”

“Helpful and allows you to target exactly what you want to.”

“It has really given me more confidence in my ability to control my IBS and to be in control of my life.”

“Very positive indeed. I am now able to relax in a way I never thought would be possible again. To gain the control and pain reduction I have achieved so far was beyond my expectations. A real boost to myself worth and confidence.”

“I now feel like I have more control over my stomach and not having to rely on tablets as much as I have been in the past. First time in over 10 years that I have been on a plane and not had to take Imodium is a massive step for me!”

“I would highly recommend it. It helps train the mind in a certain way so a person is able to control emotions and feelings in a better way.”

“Eye opening and very enjoyable. I feel it has made me realise I can relax and can have control over my symptoms. Still have work to do to keep it up but feel the most positive about my IBS than I have in years.”

“I was sceptical about hypnosis; my hypnotherapy sessions had a lasting impact, wonderful, insightful and helpful. Worked even better than I expect, my entire mind-set feels changed not only about IBS but about myself and my feelings in general.”

“Surprised by how relaxing and de-stressing it all was. I think (the therapist) actually calmed down the Hurricane at least for a while!”

“With hypnosis you have the power to train your mind into thinking differently and seeing things in a different light i.e. how we feel and how it affects the way our body works.”

“Extremely positive in physical emotional rational and spiritual aspects of my life. A much greater understanding of how mind and lifestyle affects my condition as well as inherited sensitivities.”

“This hypnotherapy programme has certainly helped me to relax and had a beneficial effect on my digestive system. The whole experience was different to any treatment that I had before.”

“Very positive. I can conceive nothing but benefits for participants with few or no costs. It was cathartic discussing health problems with a sympathetic health professional. This in itself is a rarity in my considerable experience. The benefits of hypnotherapy have been documented for centuries and speak for themselves.”

“Everything has been good but one thing that has helped me a lot with exam stress is visualising the problem in your head and moving it away.”

“I loved it and I’ll miss it! I particularly enjoyed [the hypnotherapist’s] words of wisdom before and after the hypnotherapy sessions. I honestly think I benefit as much from those as I did the hypnotherapy. Both things in combination changed the way I think for good.”

“Very interesting. Very relaxing. Made me realise a lot of my coping mechanisms were bad and harmful to my health. I can help manage my pain, mind over matter.”

“Breathing, focussing, healing, getting rid of useless negative thoughts. Connecting with yourself.”

“Amazed. I want to make others believe.”

“The sessions have transformed my feelings of hopelessness about my IBS.”

“Extremely helpful and useful - made me see things from a different perspective and feel much more positive. Reduced stomach pains and taught how to gain some control”

“Certainly worthwhile, given me a new perspective on my thought processes, my need for the “refreshment time” and appreciation for hypnotherapy.”

“He [the hypnotherapist] gave me hope in the future and not to give up and he believed in me when the doctors didn’t. It helped me relax and find some inner rest.”

“Re-enforcing the knowledge that a calm, relaxed mind can powerfully control how the body functions.”

“It was really helpful and relaxing, something I cannot normally do. Good to speak to somebody who really understood me. Helped me see different point of views.

“Excellent experience. Would recommend to fellow IBS sufferers. Helped control symptoms, enables me to relax and reduce anxieties. Has extended my knowledge of IBS, how to manage it, food intolerances etc… […] Was a very holistic programme and took into account all aspects of the IBS and my other chronic illness. Excellent !!!”

“The thinking about how body works, body calls – Have much better understating of how my body works and my hypnotherapist have a massive knowledge.”

“I honestly believe it was a bit of a life saver. [My hypnotherapist] was lovely very kind and brilliant at her job. I looked forward to our treatment and feel like I can move forward in life with more confidence and comfort.”

“After being dismissed and ignored over 25 years […], from many GPs and hospital consultants, not understanding how much it controlled and ruined my life, this worked so well it speaks for itself. This has changed my life.”

“Calming, supportive, fabulous.”

“Very worthwhile. Would advise anyone to try it. A positive, pleasant experience.”

“Extremely valuable and life changing health treatment which I feel extremely lucky to have been able to access. Helped me in many ways (forms I did not expect) such as how I interpret and manage life events and how I can improve my IBS by understanding this.”

“From day one, I saw a difference.”

“Anybody who thinks this therapy can’t work must try it first, as all pain killers and other meds did nothing, but the therapy is the only thing that made a difference.”

“A very positive, relaxing and uplifting experience, which has given me ‘my life back’ quite literally. A deeper understanding of my inner self and what I can achieve.”

“A huge thank you […] for really picking me up and putting me back on my feet again. I have been an IBS sufferer for 20 years so I am really grateful.”

“Enjoyed every moment of it and very grateful to have been given this opportunity.”

“My experience was extremely positive. The process itself has also been very beneficial, teaching me how to relax and control my symptoms.”

“The experience was enlightening and enjoyable. The hypnotherapy gave me a deep relaxation never experienced previously and I looked forward to hearing the calming voice of my tutor at each session as he enabled me to completely transcend into a peaceful state of tranquillity I have never realised existed!”

“Very pleasant sessions more relaxed and always positive. Enjoyed attending feel that the course of 12 sessions has given me back control of my IBS, life changing.”

“Educational, relaxing, learning to take time out to think and calm your mind in order to control and manage the pain and symptoms better when it occurs.”

“I’m always lost for words when trying to tell somebody how incredible hypnotherapy has been for me. Honestly, the best thing I could have done.”

“It’s a great experience. It helped me so much, literally changed my life. It is something amazing that relaxes you to the max and you feel incredible after it.”

“Welcoming, relaxing and always one of the best parts of my week.”

“It was great, felt at ease from the start to finish and felt improvement from first session.”

“Didn’t find anything not effective, felt could utilise all input from sessions.”

“The treatment provided me with a range of lifelong strategies to help me manage my condition. My experience has been extremely positive, thanks to the expertise of your hypnotherapist and the kindness of your office staff.”

“Positive, pleasant, extremely successful. I am now able to manage my condition without pointless drugs for IBS. In conjugation with the initial stripping back the diet and identifying key sources, food studs causing flare ups, my IBS is under control.”

“I have found [my hypnotherapist] extremely helpful, friendly and professional and enjoyed all the sessions with her. She has the gift of making you feel special and important for the time you spend together and this contributes greatly to the value and effectiveness of each sessions. Thank you!”

“I really enjoyed it and feel very grateful for this experience. Hypnotherapy has helped me cope with my symptoms and give me techniques to relax my stomach.”

“I would describe my overall experience as an eye opener as it is surprising how much hypnotherapy can actually help and not just with IBS but with everyday life too. My experience has been amazing I have loved every minute of it and I’m sad it’s come to an end but I’ll definitely be carrying it on in life with me.”

“Feel it could be useful to many more people with other conditions.”

“You can get a lot of help to live your life and cope with challenges.”

“2016 was a disastrous year, three deaths in my family, the start of my bowel problems followed by endless problems with medics. […] My hypnotherapist changed all of that. I had not realised I had been so down until I started to resurface again.”

“It has been a very positive experience. I always looked forward to my weekly session. They are special “me time”. It has given me a tool which I will benefit from for the rest of my life.”

“I’ve enjoyed it and I believed it’s helped me an awful lot because I walked to school almost every day and I’ve only been off once or twice while having it done which would have been very unusual before I started hypnotherapy.”

“Overall, it has been an experience that has changed my life.”

“All, the hypnotherapy and ongoing support and gradually being believed and listened to, offered help made a huge difference.”

“Brilliant, sad to finish treatment, feel like it’s been a new life.”

“Absolutely amazing…. I can’t thank my therapist enough. He has truly made some positive changes to my life and taught me things I will remember for the rest of my life.”

“Absolutely amazing… my therapist is awesome and has given me so much tools to cope in my life which makes me overall a happier person. This therapy came at very difficult time in my life and having these weekly sessions of relaxation and exercises has made me able to cope, and has given me lifelong tools I can hopefully keep using and on top of that my pain and symptoms are overall less.”

“It was so impressed. [My hypnotherapist] went above and beyond to make sure my sessions were effective, I felt very supported throughout. It is a brilliant that this is available on the NHS.”

## Changes in perceptions

“Hypnotherapy in increasing relaxation and engaging focus. Now have much better understanding of condition and more positive view.”

“I really wasn’t sure what to expect before my treatment and now I view it as a positive experience.”

“Sceptic – before. Believer – now.”

“Gave me an even better understanding of what hypnotherapy is, and it’s not just about making people look silly. Also made me realise there is more than just medication that can treat things!”

“Made me realise I don’t need to go straight to medication when I am in pain, there other alternative that could work if I tried.”

“At first I thought I would feel vulnerable and out of control, but in truth it’s the opposite.”

“I now believe that my mental state can control physical symptoms and it proves that the mind plays a big part in certain conditions.”

“I am now a firm believer that it works and I wish it could be used in other areas of the NHS too to benefit more people.”

“Had low expectations that it could work. Now, not sure how it works but cannot hide the fact that it has.”

“Totally believe in its power to work.”

“I enjoy learning about how things like this work! I know it could be used for entertainment but also to help people. This has shown me that it is effective and doesn’t require belief as such but a commitment to relaxing and changing mind-set.”

“Always assumed it wouldn’t work for me as scared to let go. Now feel it could help me with wider things in my life like anxiety and vulvodynia etc.”

“I had a positive view of hypnotherapy before starting. However, I have discovered so much more about how it works and how to build on this.”

“I am now no longer sceptical of it working.”

“Didn’t realise how powerful it was.”

“I would recommend it to any person! My treatment was brilliant, and [my hypnotherapist] was kind, understanding and so helpful. And I honestly believe now I’ve had this treatment, I can move forward with my life. The weekly sessions were especially helpful due to their consistency.”

“Now a believer and feel like I have been given the tools to be to continue the good work.”

“The belief in the healing power of hypnotherapy has strengthened.”

“Drugs and medications are not the only treatment and answer – human mind is a great unused power.”

“Was always hopeful and entered into the treatment with an open mind but it has exceeded my expectations!”

“I had not fully appreciated how my mind could have so much control over my gut or other things.”

“I am more likely to think you should give something a chance, before you pass judgement.”

“Yes, because there is nothing to be worried about, it also suitable for everyone.”

“I now believe anyone and everyone (IBS sufferer or not) would benefit from an hour to re-boot every day.”

“That this is not a TV style hypnosis… it’s more about deep relaxation.”

“I understand it is not ‘being put to sleep’ - that you are still in control.”

“You don’t have to lose consciousness!”

“Always believed it could be helpful, but now totally convinced of the important part it plays in my treatment regime.”

“I know believe hypnotherapy to be a viable treatment for IBS and stress.”

“I am more aware of its advanced nature – less likely to confuse it with “celebrity performance” hypnotherapy.”

“You were kind enough to offer me therapy in order to help me. With this in mind and even though I was at first sceptical, I decided to embrace it and give it a chance. All I can say is that it was a good decision and the whole experience I have found to be most rewarding and helpful.”

“Yes – [surprised by] same sessions each time. Thought it would vary”

“The only surprise I had is that the hypnotherapy sessions are always the same. I thought it would be different each time.”

“[Surprised by] having no concept of time while undergoing the session.”

“I expected some invasive procedure.”

“[Surprised by] how effective hypnotherapy can be! I didn’t know that it would be something I could do at home.”

“Surprised at how effective it was in such a short amount of time.”

“I have been pleasantly surprised. I did not know what to expect before treatment commenced, but it has been a much more relaxed affair than I anticipated.”

“Surprised it worked. Pleased it is available on NHS.”

“[Surprised by] it gave me an expected feeling of confidence.”

“[Surprised by] how far we can go into deep relaxation.”

“[Surprised by] how good I felt after it. The hour spent taking timeout “reprogramming” made all other hours in the day more productive.”

“Yes I did not think that I would be able to get into such a relaxed state.”

“Surprised by normality of it, hypnotherapy is different to stage performance.”

## Therapy downsides

“The repetitiveness made me zone out by the 4th session as I knew the pattern and did not relax as much. The change of descriptions/words by the hypnotherapist then helped.”

“I am not sure I understand how to apply this “few weeks” of positive improvements to a condition that I’ve suffered with for 6+ years. What if the worst symptoms recur??”

“That the hypnotherapy session was the same every time. My previous experiences had a variety of recordings.”

“A lot to squeeze into a working day with family commitments.”

“Imaging. Very poor and clashes with experience […]. Really spooked by image of me and my subconscious meeting and merging.”

“The daily practice, whilst this is important it’s very hard to dedicate 1 hour a day everyday”

“I did not fully understand the reason for so many sessions. Once I had the tape I could just practice by myself thereby giving someone else a chance to benefit. Although I do feel top up sessions every few months would be beneficial.”

“Sometimes I struggled with the visualisation but I am sure that will get better the more I practice.”

“I found that I could relax and listen during the hypnotherapy sessions but not being able to switch off whilst listening to the CDs at home.”

“Actually helping me move my bowels. Pain still keeping me awake at night because of blockage.”

“I have been very positive about the service, however I don’t feel it has helped my gut, symptoms have not improved.”

“Only one narrative to listen to – a change half-way through would have encouraged more concentration (less chance for complacency).”

“I do not feel that some of the repetition of the hypnotherapy words were appropriate to me, I wished that it could have been more person centred and words specifically tailored to my symptoms.”

“I feel that the treatment was all effective, but as my personal problems are ongoing and my IBS is mostly stress related, until my personal problems and ill health improve it will be a slow progress.”

“I cannot yet bundle up my bad thoughts about pain and bowel movement. They are clear as ever. The symptoms are still with me. I am braver about things but not totally.”

“Not being convinced I was ever hypnotised or fully absorbed in what [my hypnotherapist] was saying. Sometimes background noises but not often.”

## Miscellaneous (not able to categorise in the paper)

“[Future] hopeful. I would have liked the sessions to continue for longer as feel I am getting much better but still have a way to go.”

“I now feel I have a future. Which means I can go forward with more confidence, easier interactions with others, closer relationship with my husband, a regained ability to enjoy life!”

“A new journey ahead, been guided on my abilities to learn a new life without my IBS taking control.”

“Resigned to dealing with this miserable condition knowing it will have to be managed but feeling I have some tools to help me.”

“I see the future as being much more hopeful and positive than before the treatment. I am now much more optimistic.”

“[Future] in a positive, exciting way with more understanding and control of what had become a life changing/ inhibiting condition.”

“For now, the future means tomorrow. These are outstanding problems to be resolved still.”

“Not sure much will change for me personally – currently relapsing into depression for the 100^th^ + time, it’s exhausting!”

“Probably much the same. I am trying to be more positive, physical symptoms just as bad.”

“By continuing to use my hypnotherapy recordings at home, I can see the improvements to my digestive system also continuing.”

“I am just sorry it ended because I feel vulnerable again.”

“An ongoing war which I seem to be losing. I win battles regularly but I am losing battles more than I’m winning them in recent years hence why I am losing the war. I desperately want my life back. I want a future, I want security etc. I am trying hard but I feel like I am not progressing.”

“[Future] hopefully, IBS free. If it recurs, I will go back to listening to the recordings more frequently.”

“Very positive. My friends and colleagues always say how I am a different person even after my first session, I was so much more happy and full of life.”

“[Future] being able to pursue my career more without having to be anxious about needing the toilet in certain situations.”

“[Future] similarly to when I started treatment, worried if I will ever get better.”

“[Future] possibly better but I would have to keep listening to the recordings at regular intervals to re-enforce calmness.”

“I have to cope with my IBS, but in order my gut to work properly other conditions need to get better too. I see my body as a whole.”

“[Future] with hope and looking forward to getting married now.”

“Ongoing battle with IBS. Have picked up some tools, please to be able to contact my hypnotherapy regarding IBS.”

“[Future] with sunglasses on! Seriously – whatever happens I’ll have more control.”

“[Future] positive, yes I will have up and downs (and have had during this journey, yet as long as I continue to practice and listen to my CD I don’t see why things won’t continue to progress.”

“I feel hypnotherapy will be a coping strategy that I will always use.”

“[Future] hopeful – can’t give a final answer unless the results continue.”

“[Future] a lot brighter – more in control of my gut, more confident to go into situations where before I would have avoided: no loos, bus trips, train journeys, long queues etc.”

“More optimistic balance, being regained IBS not dominating all I do, not fully there yet but on the way. Confidence in taking part in normal activities.”

“I feel a lot more in control and positive about this condition. I believe that in the right frame of mind I could see a cure, I came to understand that my symptoms were not really that bad compared to others.”

“I understand that I have just begun learning how to use the hypnotherapy techniques and as I continue to practice they will help me to further improve in managing my IBS.”

“How scary to answer! I will keep up the home practice and stick to medication regime. Feel this gives me solid tools to manage IBS. Currently doing really well so want that to last!”

“Moving forward very slowly. One day I will find the answer.”

“I see my future in a better light then I did before because now I know how to manage and control my pain and stress levels and before this I would stress at anything even little things and I would go straight the tablets to stop the pain. I don’t need to now.”

“I’d really value some pointers/signposts in terms of who/how I engage with going forwards. In say a few months/years if symptoms recur I want to avoid a situation where I am back into a non-specialist GP dialogue. I.e. what is the after-programme care?”

“The only thing I could mention here is that such a brilliant programme deserves better facilities and a 21^st^ century photocopier!”

“My low scores do not reflect my therapist’s input. It was an impossible task for much improvement at this stage due to numerous medical issues. Perhaps one day in the future I may be allowed funding for it to be repeated once this settles down?”

“Overall, it was positive but the constipation issue is not resolved without laxatives. And this is still a thing I need to address because it is anxiety relaxed. First thing in the morning.”

“I wish I had come 10 years ago when I was first offered the experience.”

“Thank you to all those involved in face to face treatment planning and development of this service and its implementation.”

“Thank you! I love the NHS and feel very privileged to have accessed this treatment. Very grateful to all.”

“Professor Whorwell and [my hypnotherapist] have been excellent. From the first day of meeting them both, I felt more positive about my life. I would like them both on speed dial.”

“Thank you NHS for helping me get better!”

“Hypnotherapy has proven to be a life changing treatment that has benefitted me in revolutionary ways!”

**Table S1: Frequency of adjectives used by patients to describe feelings about hypnotherapy before starting treatment**

| **Word** | **Frequency** | **%** | **Word** | **Frequency** | **%** |
| --- | --- | --- | --- | --- | --- |
| **Hopeful** | 53 | 18.2 | **Enthusiastic** | 5 | 1.7 |
| **Sceptical** | 37 | 12.7 | **Doubtful** | 5 | 1.7 |
| **Excited** | 36 | 12.3 | **Pleased** | 4 | 1.4 |
| **Nervous** | 23 | 7.9 | **Inspired** | 4 | 1.4 |
| **Intrigued** | 21 | 7.2 | **Negative** | 3 | 1.0 |
| **Open-minded** | 17 | 5.8 | **Concerned** | 3 | 1.0 |
| **Optimistic** | 16 | 5.5 | **Determined** | 2 | 0.7 |
| **Interested** | 15 | 5.2 | **Helpless** | 1 | 0.3 |
| **Positive** | 9 | 3.1 | **Downhearted** | 1 | 0.3 |
| **Apprehensive** | 8 | 2.7 | **Pensive** | 1 | 0.3 |
| **Desperate** | 6 | 2.1 | **Embarrassed** | 1 | 0.3 |
| **Scared** | 6 | 2.1 | **Accepting** | 1 | 0.3 |
| **Anxious** | 6 | 2.1 | **Withdrawn** | 1 | 0.3 |
| **Unsure** | 5 | 1.7 | **Expectant** | 1 | 0.3 |

**Table S2: Frequency of adjectives used by patients to describe feelings about hypnotherapy after treatment**

| **Word** | **Frequency** | **%** | **Word** | **Frequency** | **%** |
| --- | --- | --- | --- | --- | --- |
| **Relaxed** | 54 | 20.3 | **Helpful** | 3 | 1.1 |
| **Calm** | 40 | 15.1 | **Enlightened** | 3 | 1.1 |
| **Positive** | 29 | 11.0 | **Believer** | 2 | 0.8 |
| **Happy** | 17 | 6.4 | **Supported** | 2 | 0.8 |
| **Grateful** | 15 | 5.7 | **Empowered** | 2 | 0.8 |
| **Relieved** | 13 | 4.9 | **Effective** | 1 | 0.4 |
| **Hopeful** | 8 | 3.0 | **Unsure** | 1 | 0.4 |
| **Energised** | 7 | 2.6 | **Lightheaded** | 1 | 0.4 |
| **Optimistic** | 6 | 2.3 | **Potential** | 1 | 0.4 |
| **Satisfied** | 6 | 2.3 | **Focussed** | 1 | 0.4 |
| **In control** | 5 | 1.9 | **Rejuvenated** | 1 | 0.4 |
| **Better** | 5 | 1.9 | **Useful** | 1 | 0.4 |
| **Disappointed** | 5 | 1.9 | **Mindful** | 1 | 0.4 |
| **Surprised** | 4 | 1.5 | **Remarkable** | 1 | 0.4 |
| **Encouraged** | 4 | 1.5 | **Healthy** | 1 | 0.4 |
| **Unhappy** | 4 | 1.5 | **Exhausted** | 1 | 0.4 |
| **Uplifted** | 4 | 1.5 | **Stable** | 1 | 0.4 |
| **Motivated** | 4 | 1.5 | **Disorientated** | 1 | 0.4 |
| **Amazed** | 4 | 1.5 | **Whole** | 1 | 0.4 |
| **Rested** | 4 | 1.5 | **Pain free** | 1 | 0.4 |

**Table S3 – Alternative situations for which patients found hypnotherapy helpful.**

| **Activity of daily life** | **Frequency** | **%** | **Activity of daily life** | **Frequency** | **%** |
| --- | --- | --- | --- | --- | --- |
| **Relaxing** | 40 | 16.3 | **Social events** | 3 | 1.2 |
| **Breathing** | 30 | 12.2 | **Exams** | 3 | 1.2 |
| **Dealing with anxiety** | 24 | 9.8 | **Self Esteem** | 3 | 1.2 |
| **Dealing with stressful situations** | 24 | 9.8 | **Mindful** | 2 | 0.8 |
| **Staying Calm** | 23 | 9.3 | **Putting things in perspective** | 2 | 0.8 |
| **Sleep** | 18 | 7.3 | **Coping with surgery** | 2 | 0.8 |
| **Listening to CD** | 16 | 6.5 | **Getting others to understand the benefit** | 1 | 0.4 |
| **Controlling Aches and Pains** | 12 | 4.9 | **Reflecting** | 1 | 0.4 |
| **Preventing anger** | 10 | 4.1 | **Physical well-being** | 1 | 0.4 |
| **Driving** | 6 | 2.4 | **Quitting smoking** | 1 | 0.4 |
| **Meditating** | 5 | 2.0 | **Feeling in control** | 1 | 0.4 |
| **Confidence** | 4 | 1.6 | **Slowing down** | 1 | 0.4 |
| **Focussing** | 4 | 1.6 | **Living in the moment** | 1 | 0.4 |
| **Removing negative thoughts** | 4 | 1.6 | **Relating to people** | 1 | 0.4 |
| **Family life** | 3 | 1.2 |  |  |  |
